# Strategies for determining kinship in wild populations using genetic data

**DOI:** 10.1002/ece3.2346

**Published:** 2016-07-29

**Authors:** Veronika Städele, Linda Vigilant

**Affiliations:** ^1^ Department of PrimatologyMax Planck Institute for Evolutionary AnthropologyDeutscher Platz 6D‐04103LeipzigGermany

**Keywords:** Genetic relatedness, microsatellites, next‐generation sequencing, parentage analysis, sibship reconstruction, single‐nucleotide polymorphisms

## Abstract

Knowledge of kin relationships between members of wild animal populations has broad application in ecology and evolution research by allowing the investigation of dispersal dynamics, mating systems, inbreeding avoidance, kin recognition, and kin selection as well as aiding the management of endangered populations. However, the assessment of kinship among members of wild animal populations is difficult in the absence of detailed multigenerational pedigrees. Here, we first review the distinction between genetic relatedness and kinship derived from pedigrees and how this makes the identification of kin using genetic data inherently challenging. We then describe useful approaches to kinship classification, such as parentage analysis and sibship reconstruction, and explain how the combined use of marker systems with biparental and uniparental inheritance, demographic information, likelihood analyses, relatedness coefficients, and estimation of misclassification rates can yield reliable classifications of kinship in groups with complex kin structures. We outline alternative approaches for cases in which explicit knowledge of dyadic kinship is not necessary, but indirect inferences about kinship on a group‐ or population‐wide scale suffice, such as whether more highly related dyads are in closer spatial proximity. Although analysis of highly variable microsatellite loci is still the dominant approach for studies on wild populations, we describe how the long‐awaited use of large‐scale single‐nucleotide polymorphism and sequencing data derived from noninvasive low‐quality samples may eventually lead to highly accurate assessments of varying degrees of kinship in wild populations.

## Why Determine Kinship in Wild Animal Populations?

In many social species, members of one sex disperse, while members of the philopatric sex live in close proximity to kin and nonkin. Distinguishing between close relatives and unrelated conspecifics allows individuals to obtain direct or inclusive fitness benefits by biasing affiliative or coalitionary behaviors toward relatives while avoiding inbreeding and competition with relatives (Hamilton 1964). For example, recent studies of wild populations have demonstrated an effect of kinship on allonursing in cooperative breeders (MacLeod et al. 2013), identified kin biases in association (Bercovitch and Berry 2013) and affiliation (Widdig et al. 2016), and shown parallel dispersal of kin (Wikberg et al. 2014) and inbreeding avoidance (Sanderson et al. 2015). Association and close social relationships among relatives may provide adaptive benefits by improved reproductive success through increased longevity or offspring survival (König 1994; Viblanc et al. 2010).

Beyond dyadic social relationships, knowledge of the population‐wide distribution of pairs of kin and nonkin can be used to identify dispersal patterns (Van Noordwijk et al. 2012) or reproductive skew (Vigilant et al. 2015). Several studies have used kinship analyses to characterize mating systems of wild populations as monogamous (Huck et al. 2014), polyandrous (Barth et al. 2014), or polygynous (Muralidhar et al. 2014), identified extra‐pair parentage in socially monogamous species (Barelli et al. 2013) or cases of adoption and cuckoldry (Stiver et al. 2012). Moreover, at the population level, even members of solitary species may derive benefits from kin biases by avoiding inbreeding (Metzger et al. 2010) or competition among relatives (Lizé et al. 2006) or by occupying territories next to kin which may lead to reduced aggression (Bradley et al. 2004). Furthermore, dyadic kinship information can be used to estimate the heritability of traits (Dubuc et al. 2014). From a practical perspective, kinship analyses can be applied to inform conservation efforts such as the breeding and stocking management of endangered fish populations (O'Reilly and Kozfkay 2014).

Studies of kinship in the wild are often preferable over studies in captivity because aspects such as the kinship structure in the population, dispersal, mating as well as kin‐biased behavior may be strongly altered under captive conditions. However, analysis of kinship patterns can be challenging in wild populations. Maintenance of long‐term field sites with individually identified animals and reconstructed multigenerational pedigrees is challenging (Clutton‐Brock and Sheldon 2010). A long‐standing goal in kinship studies has therefore been to assess kin relationships by the use of genetic analysis, which has typically employed microsatellite genotype analysis of DNA derived from noninvasive samples.

## Genes or Genealogy?

**Figure 1:**
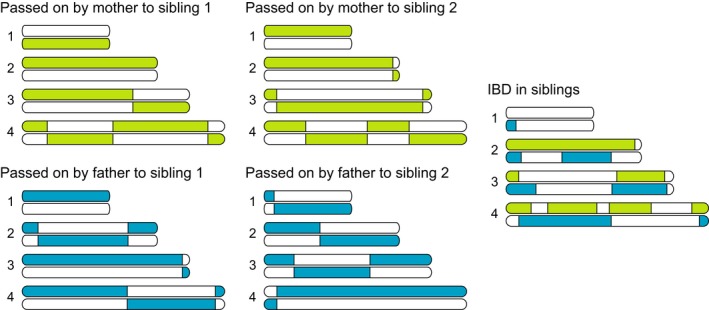
Schematic of four chromosome pairs, showing the parental origins of segments of the genome shared identical by descent (IBD) by a pair of full siblings. Green segments of the genome are passed on to each offspring by the mother, and blue segments are passed on to each offspring by the father. Due to crossover events, parts of either chromosome can be passed onto the offspring.

Comparing genotypes of different individuals and classifying them into kinship categories such as “sister” or “cousin” are difficult. This is because genetic relatedness is a continuous parameter determined by the proportion of the genome shared between two individuals by descent from a common ancestor and, particularly if inferred from a limited number of markers, does not necessarily correspond to theoretical expectations based on the categorical pedigree relationship for a given dyad (Blouin 2003). The segregation of pairs of chromosomes during the first meiotic cell division as well as chromosomal recombination is stochastic processes leading to large variation in the amount of the genome that is identical by descent between two relatives, with the exception of parent–offspring and monozygotic twins (Fig. 1; Rasmuson 1993). For example, while full siblings share on average 50% of their genome, some may share considerably less or more (e.g., Visscher et al. 2006; Fig. 1) the variance being dependent on the number of chromosomes and their crossover rates (Hill and Weir 2011). Therefore, although they are generally strongly correlated, *pedigree relatedness* or *kinship* and *genetic relatedness* or *realized relatedness* is conceptually and often empirically different.

Pedigree estimates of relatedness may be inaccurate because they require the assumption that founders are outbred and unrelated. In combination with the increased ability to accurately determine realized relatedness, this has led many to question the usefulness of relatedness derived from pedigrees, particularly in the context of heritability and inbreeding (Gay et al. 2013; Speed and Balding 2014; Kardos et al. 2015; Wang 2015). Recent human studies even use the actual genetic similarity of large numbers of unrelated individuals, instead of close relatives, to estimate heritability and predict phenotypes. This use of unrelated individuals reduces the variance in inferred heritability among dyads and increases the possibility of pinpointing the heritability of a trait to specific genomic regions (Speed and Balding 2014). In theory, the same principal could be used to investigate kin recognition in species which recognize kin via phenotype matching by correlating genetic similarity with biases in behavior as well as to identify the genetic regions involved. The more genetically similar two individuals are, the more likely they are to share alleles for the genes involved in kin recognition by phenotype matching. According to Hamilton's (1964) rule, we would thus predict that individuals prefer more genetically similar individuals independent of their categorical kinship. Yellow baboons, for example, likely recognize paternal kin via a combination of social familiarity and phenotype matching (Smith et al. 2003), but strong social bonds also exist among unrelated individuals (Silk et al. 2006). One could thus hypothesize that preferred unrelated social partners are chosen based upon genetic similarity due to “misdirected” kin recognition by phenotype matching. For yellow baboons, other factors, such as rank or age similarity, almost certainly have larger effects than genetic similarity in influencing the choice of social partners among unrelated individuals, so that thousands of individuals might be necessary to obtain sufficient power to detect any effects of genetic similarity on kin biases (Silk et al. 2006; Visscher et al. 2014). In an experimental design, juveniles of Atlantic salmon and brook trout preferred kin with whom they shared both alleles for an MHC class II gene to kin with whom they shared no alleles and preferred nonkin sharing both alleles to nonkin sharing no alleles (Rajakaruna et al. 2006). In this study, the influence of a candidate gene on kin recognition was investigated. Generally however, if a limited number of markers are used to determine genetic relatedness, and these markers are not by chance linked to genetic regions involved in kin recognition, pedigree relatedness should more accurately represent the genome‐wide sharing of alleles than genetic relatedness and may then be the more accurate predictor of kin bias. Analogous comparisons of marker‐ and pedigree‐based heritability estimates show that thousands of single‐nucleotide polymorphisms (SNPs) are necessary to estimate heritability with the same accuracy as when using pedigree relatedness (Gay et al. 2013; Bérénos et al. 2014). Such numbers of SNPs are still unavailable for most studies of nonmodel organisms which particularly for wild populations often rely upon poor‐quality DNA derived from noninvasive samples and consequently employ analyses of relatively small numbers of microsatellite loci (Box 1).

Box 1 **Genetic marker systems and noninvasive sampling.**

**Figure B1:**
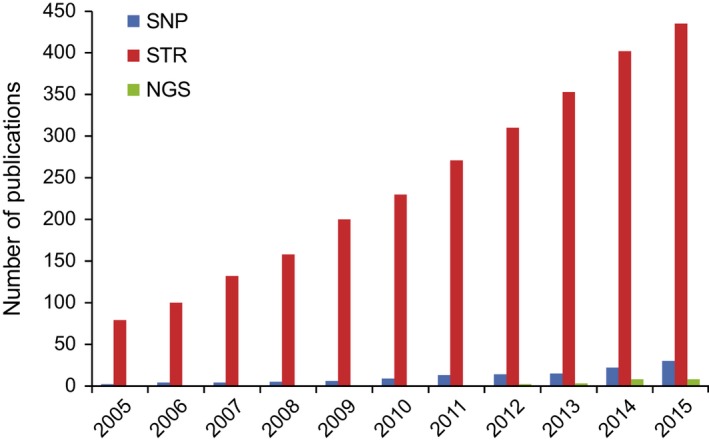
ISI Web of Knowledge cumulative search results per year Search words: “wild population” and “microsatellite” (STR) or “SNP” (single‐nucleotide polymorphism) or “next‐generation sequencing” (NGS), excluding “plant.”

Studies of wild animals typically rely upon noninvasive samples such as hair (Morin & Woodruff 1992), blow (Frère et al. 2010), food wadges (Hashimoto et al. 1996), feathers and egg membranes (Pearce et al. 1997), shed skin (Villarreal et al. 1996), urine (Hayakawa & Takenaka 1999), or fecal samples (Höss et al. 1992). Although the DNA extracted from these samples is usually degraded and contains low proportions of endogenous DNA, accurate microsatellite genotypes can be obtained if extensive replication is performed (Taberlet & Luikart 1999). First described in the 1980s, microsatellites (STRs) are tandem repeats of short sequences and have long been the most common markers used in studies of wild populations (Fig. B1) with single‐nucleotide polymorphisms (SNPs), single base‐pair differences between the genomes of two individuals of a species, and next‐generation sequencing being less commonly used. The advantages and disadvantages of microsatellites and SNPs for population genetic applications in general have been extensively reviewed (Morin et al. 2004; Guichoux et al. 2011).

**Advantages of STRs in kinship analyses**

**Figure B2:**
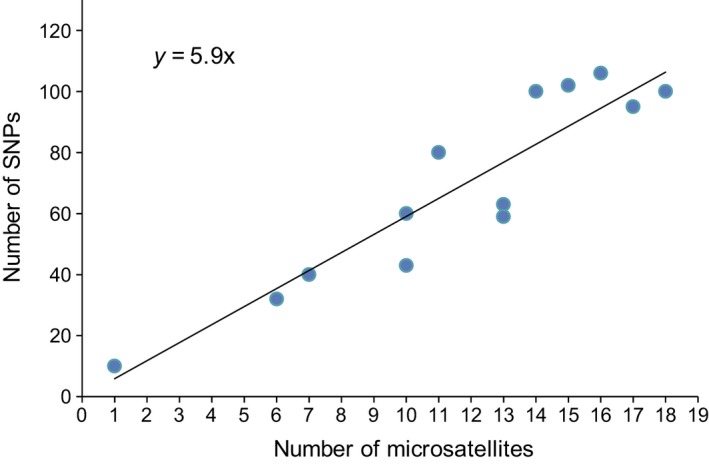
Relationship between the number of microsatellites (STRs) and the number of single‐nucleotide polymorphisms (SNPs) when both marker systems perform equally well in kinship analyses. Data are simulated data or empirical population data (Table S1).

- Highly polymorphic.
- High cross‐species amplification success (e.g., Buschiazzo & Gemmell 2010).
- Sibship reconstruction possible according to the 4‐ and 2‐allele property (Berger‐Wolf et al. 2007).
- Generally high power for kinship analyses; ˜6× the power of SNPs (Fig. B2).

**Advantages of SNPs in kinship analyses**

- Biallelic: Few genotypes necessary to accurately estimate allele frequencies.
- Lower and predictable mutation rates (Ellegren 2004).
- Shorter fragments amplified: Greater amplification success from degraded DNA (Campbell & Narum 2009).
- As many loci have to be typed, the resulting genotypes may be more representative of the entire genome.
- Software for the analysis of genetic marker data is increasingly developed for SNP data only.

In contrast to phenotype matching, kin recognition mediated by familiarity or contextual cues is independent of genetic relatedness, but dependent on pedigree relationships and the resulting spatial and temporal association of individuals. These include a close association between mother and offspring in species with maternal care, close association of littermates at a young age which usually are maternal or full siblings, or age proximity which could be used as a cue for paternal relatedness in species for which male reproductive skew leads to cohorts of paternal siblings (Widdig 2013). Cross‐fostering experiments have shown that individuals bias their behavior toward familiar nonkin over unfamiliar kin (reviewed in: Mateo and Holmes 2004). In most mammals, individuals may recognize their mothers, but may not bias their behavior toward other individuals having the same degree of genetic relatedness (full siblings, father–offspring), and thus, the pedigree relationship, and not the degree of genetic relatedness, is informative with regard to kin bias. For example, chimpanzee males bias affiliative and cooperative behaviors toward maternal, but not paternal brothers despite a nominal relatedness coefficient of 0.25 for both kinds of relatives (Langergraber et al. 2007). As genetic similarity, particularly when determined from a limited set of genetic markers, does not distinguish among these different kinds of kin and variance for even the same type of kin is high, the indiscriminate inclusion of the type of kin that cannot be recognized will lower the correlation of genetic relatedness and kin bias. Therefore, if individuals recognize kin through kinship‐correlated familiarity or contextual cues, pedigree kinship and not genetic relatedness will be the best predictor of kin bias, suggesting that even in the genomic era, knowledge of pedigree relationships can be useful. As detailed in the following sections, even the small sets of genetic markers typically available for studies of wild populations can be used to make inferences on kin relationships.

## Assessing Parentage

**Figure 2:**
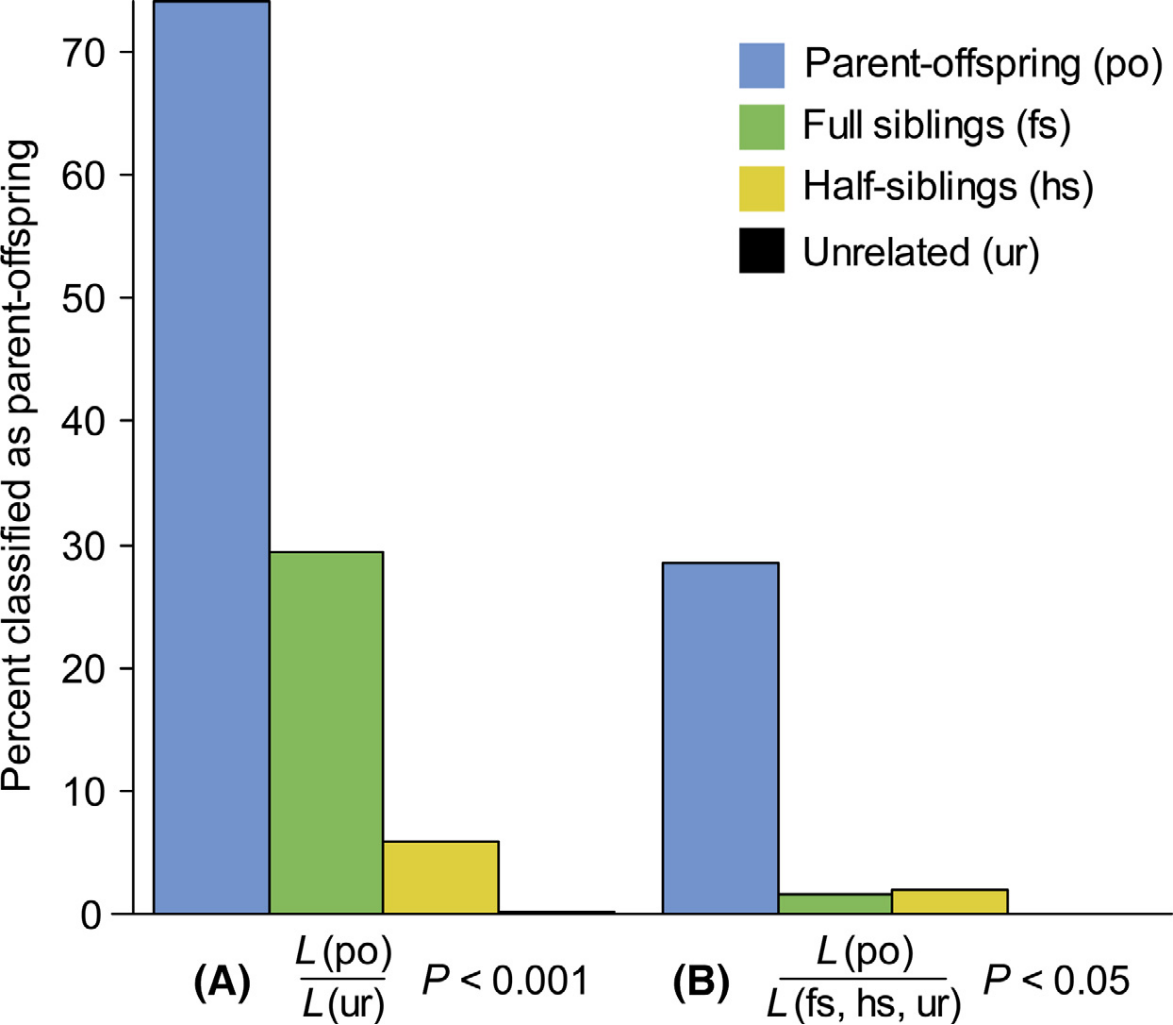
Proportion of kin (mis)classified as parent–offspring in likelihood analyses of parentage. The likelihood ratio value is the likelihood (*L*) of the alternative hypothesis of parent–offspring relationship over the likelihood of a null hypothesis of (A) no relatedness or (B) a complex null hypothesis simultaneously considering full siblingship, half siblingship, and no relatedness. Even when using conservative *P*‐value, misclassifications occur (A) while testing a complex null hypothesis (B) reduces the number of misclassifications of other kin categories as parent–offspring but more than halves the proportion of true classifications. Sets of 1000 dyads per kinship category (po: parent–offspring, fs: full siblings, hs: half siblings, ur: unrelated) were generated in KINGROUP v2 (Konovalov et al. 2004) using ten loci with five equifrequent alleles per locus. Likelihood analyses were conducted in KINGROUP v2. *P*‐values were generated through 1,000,000 permutations.

Parent–offspring relationships can be determined with higher confidence than other relationships because, with the exception of instances of germline mutations or genotyping error, the parent and the offspring *must* share at least one allele at every locus. In many wild species, parental care, typically by the mother, easily identifies one likely parent. Direct comparison of mother, offspring, and potential father genotypes, if sufficiently variable, may directly reveal parentage relationships if all candidate parents were perfectly sampled. However, analysis in a statistical framework that allows for the consideration of error rates, proportion of candidate parents sampled, and other factors can aid in assessing the confidence of the assignments (e.g., CERVUS (Marshall et al. 1998)), FRANz (Riester et al. 2009), KINGROUP (Konovalov et al. 2004)). For example, testing for parentage in a likelihood framework assesses the significance of the likelihood ratio of a dyad, that is, the likelihood that the dyad has a certain relationship given its patterns of allele sharing (e.g., parent–offspring) over the likelihood that the dyad has alternative relationships (e.g., unrelated) (Fig. 2).

Parentage analysis becomes markedly more challenging in situations where neither parent is known by observation. Essentially, the same principal of shared alleles and exclusion can be applied, but the assignment becomes much more complicated as the identities of the maternal and paternal alleles in the offspring are unknown. Several different approaches have been devised to assign parentage if few or no parent–offspring relationships are known or several sires cannot be excluded (Jones et al. 2010; Harrison et al. 2013). Generally, assignment error increases with an increasing number of candidate parents, but decreases the greater the proportion of candidate parents sampled (Marshall et al. 1998; Harrison et al. 2013). Assignment error also depends on the presence of other categories of kin in the sample. This is because a nonparent relative of either the offspring or a true parent, particularly one related to the offspring or parent at a level of 0.25 or higher, is likely to be misclassified as a parent (Thompson and Meagher 1987; Marshall et al. 1998; Olsen et al. 2001; Fig. 2).

Thus, despite the unique genetic relationship between parent and offspring, false‐positive and false‐negative assignments are to be expected in parentage analyses. Given that unrelated individuals are highly unlikely to be classified as parent–offspring (Fig. 2) and that knowledge of parentage is likely used to assess a behavioral or ecological hypothesis, it might be acceptable that some putative parent–offspring dyads are actually not parent–offspring, but otherwise closely related.

Yet, in long‐term studies, for which one parent is known by observation and the other genetically assigned, assignment error will be extremely low and continued parentage analysis can identify maternal and paternal kinship over generations and thus be used to reconstruct increasingly deep pedigrees (e.g., Van Horn et al. 2008).

## Sibship Reconstruction

In species for which the population can be expected to mainly contain groups of full and/or half siblings, sibship reconstruction is a powerful tool for identifying the related individuals. This approach is more accurate than evaluating dyads because it considers the relationships among all genotypes simultaneously (Wang and Santure 2009).

The success of sibship reconstruction generally improves with increases in the number of individuals per full‐ or half‐sib family, although full sibship may be determined with high accuracy for sibling groups as small as four (Wang and Santure 2009), but may decrease with an increasing number of families (Thomas and Hill 2002; Sheikh et al. 2008; Almudevar and Anderson 2012; Wang 2012). For example, with just four highly variable loci, 12 full‐sibling families of 760 Atlantic salmon could be accurately partitioned (Almudevar and Anderson 2012; Wang 2012). Such analyses are extremely accurate, but become less successful if dyads with a lower degree of relatedness are included; for example, the inclusion of cousins reduces the power and accuracy of the analysis (Thomas and Hill 2002; Wang 2004). Analysis of populations with complex kinship structures, such as may arise when both sexes are polygamous, can lead to prohibitively long run times and nonconvergence (Wang and Santure 2009; Wang 2012; Dexter and Brown 2013).

Such complexity, including the coresidence of different categories of close and distant relatives, may be present in social groups featuring promiscuous mating systems, small litter sizes, long life spans, overlapping generations, or immigration. Hence, approaches identifying different types of kin in groups with complex kin compositions are needed.

## Identifying the Other Types of Kin

**Figure 3:**
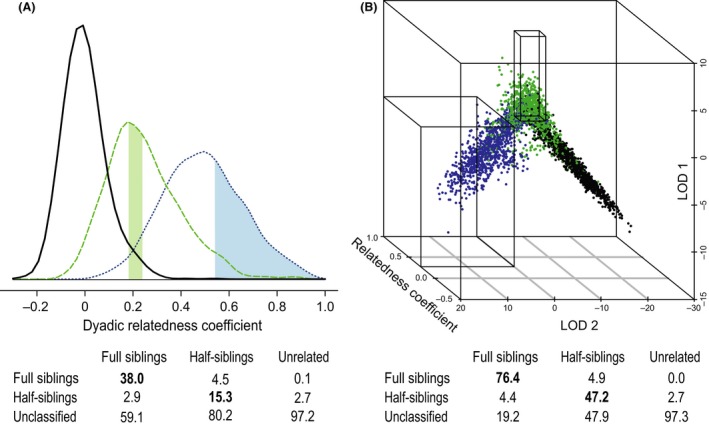
Misclassification and true classification rates per kinship category. Dyads of unrelated individuals (black), half siblings (green), and full siblings (blue) genotyped at 23 autosomal microsatellite loci were simulated in KINGROUP v2 using allele frequencies of a band of hamadryas baboons (Band 1, Städele et al. 2015). Tables indicate the percentage of true classifications (bold), misclassifications, and unclassified dyads. The top row indicates the actual relationship. Truly unrelated dyads were combined with unclassified dyads, but could also be classified by defining cutoff values. (A) Cutoff values for the relatedness coefficient (edges of the shaded areas) can be modified to achieve low misclassification rates leading to low rates of true classifications (bold). Shaded areas indicate the proportion of dyads classified as half (green) or full siblings (blue). (B) Cutoff values for relatedness coefficient and two likelihood ratios applied simultaneously (lines); log‐likelihood ratio 1 (LOD 1) hypothesis: half siblings, null hypothesis: parent–offspring, full siblings, unrelated; log‐likelihood ratio 2 (LOD 2) hypothesis: full siblings, null hypothesis: parent–offspring, half siblings, unrelated. This approach leads to similarly low misclassification rates as A) but allows for more true classifications.

One seemingly straightforward approach to determining the kin relationship of any dyad relies on the use of dyadic relatedness estimators, which gauge the amount of genetic material shared by descent between individuals. The accuracy and precision of these estimators depend on the number of markers typed, their polymorphism, allele frequency distribution, and the kin structure of the population (Milligan 2003; Csilléry et al. 2006; Konovalov and Heg 2008; Van Horn et al. 2008). Although the relatedness coefficient averaged over many dyads usually corresponds well to the expected pedigree relatedness, the previously discussed *inherent* difference between genetic and pedigree relatedness leads to overlapping distributions of the relatedness coefficient for different kinship categories (Blouin et al. 1996; Fig. 3A). This is in principal independent of *methodological* inaccuracies in genetic relatedness estimates due to the usage of a limited number of genetic markers, variance in the sharing of alleles by state, or inaccurate measures of the population's allele frequencies. Consequently, the relatedness coefficient for any dyad is an imperfect measure of that dyad's pedigree kinship, and correlations between pedigree and genetic relatedness will be imperfect. This thus holds true even if very large numbers of markers are used. For example, a study in zebra finches found a maximum correlation of 0.86 between the genetic relatedness determined with the full dataset of 771 SNPs and the pedigree relatedness of a multigenerational zebra finch pedigree. Because linkage among loci increases the variance of the estimate (Glaubitz et al. 2003; Santure et al. 2010), even a study using more than 9000 SNPs to assess the correlation between genetic and pedigree relatedness in pigs found a correlation of just 0.85 (Lopes et al. 2013).

Despite the lack of a perfect correlation of genetic relatedness with pedigree relatedness, some approaches exist to identify dyads which can be classified with confidence. For example, Blouin et al. (1996) suggested first using simulated distributions of the relatedness coefficient for certain kinship categories and then defining cutoff values to classify dyads as belonging to these kinship categories while determining consequent misclassification rates from the simulated distributions. How the cutoff values are chosen determines the misclassification rate. It is possible to thus push the misclassification rates for certain kinship categories under a desired threshold (e.g., 5%, although much lower rates might be desirable if many dyads are evaluated) by choosing narrow cutoff values (shaded areas in Fig. 3A). However, this approach leads to low rates of true classifications, that is, dyads of a certain kinship category which are correctly classified as belonging to that category, and many dyads remain unclassified because their values fall between the cutoffs (Fig. 3A).

By combining cutoffs for likelihood ratios, often expressed as the logarithm of the likelihood ratio (log odds ratios, LOD) and testing different hypotheses about the kinship status of a dyad, it may be possible to improve the resolution of such an analysis (Thompson and Meagher 1987). Additional power can be added by combining cutoffs for likelihood ratios with cutoffs for the relatedness coefficient (Langergraber et al. 2007; Städele et al. 2016; Fig. 3B). Although relatedness coefficients and likelihood ratios are strongly correlated because they are derived from the same autosomal data, they are sufficiently different so that the resolution of classifications of dyadic kinship is improved by combining them. This leads to low misclassification rates combined with high true classification rates (Fig. 3B). Cutoffs can be defined by systematically testing values which maximize the true classification rate and minimize the misclassification rate or can be empirically defined using data from dyads of known pedigree relationship or from relatives identified through pedigrees reconstructed after parentage analysis (Langergraber et al. 2007; Städele et al. 2016). Using such an approach, low misclassification rates can be achieved with a relatively small set of loci. It is important to note that even when using this combined approach, if low misclassification rates are prioritized, a large number of dyads will be unclassified because their parameter values fall between the cutoff values for different kinship categories (Fig. 3B).

A different approach to identifying dyadic kinship which also accepts unclassified dyads as a trade‐off for low misclassification rates is based on calculating *P*‐values associated with likelihood ratios and then selecting a subset of dyads by applying the false discovery rate procedure (Benjamini and Hochberg 1995) which controls for an expected proportion of type I errors (Skaug et al. 2010). The subset of dyads is then genotyped at a second set of loci, and dyads are accepted as having the hypothesized kin relationship according to a nominal level of *P*‐value associated with the new likelihood ratio value.

These approaches could theoretically be used to classify dyads beyond the second degree of kinship; however, the overlap of the distributions of measures of genetic relatedness increases with a decreasing degree of kinship, and eventually, no satisfactory trade‐off between misclassifications and correct classifications can be reached.

In sum, by accepting the limitations of certain rates of misclassification as well as the inability to classify every single dyad, it is possible to infer dyadic kinship up to the second degree for many pairs of individuals living in populations with complex kin compositions, even when using the limited numbers of autosomal markers typically available for wild populations.

## The Value of Nonautosomal Marker Data and Demographic Information

The inheritance patterns of the maternally inherited mitochondrial DNA (mtDNA), paternally inherited Y‐chromosome, and bi‐parentally inherited X‐chromosome make them powerful additions to kinship analyses using autosomal data and by identifying false‐positive assignments of kinship as well as reducing the misclassification rates (Kopps et al. 2015). Of the nonautosomal markers, mtDNA is commonly used in studies of wild populations due to a high degree of sequence identity of many of its segments among vertebrates making it an easy target for cross‐species amplification, as well as the presence of high copy numbers leading to usually good amplification from low‐quality samples. X‐ and Y‐linked loci have been less widely used and may have to be identified *de novo* for many species, but these can in principal also be genotyped using cross‐species amplification, although low levels of Y‐chromosomal variation can make it difficult to identify polymorphic Y‐linked markers (Ellegren 2003; Greminger et al. 2010).

**Figure 4:**
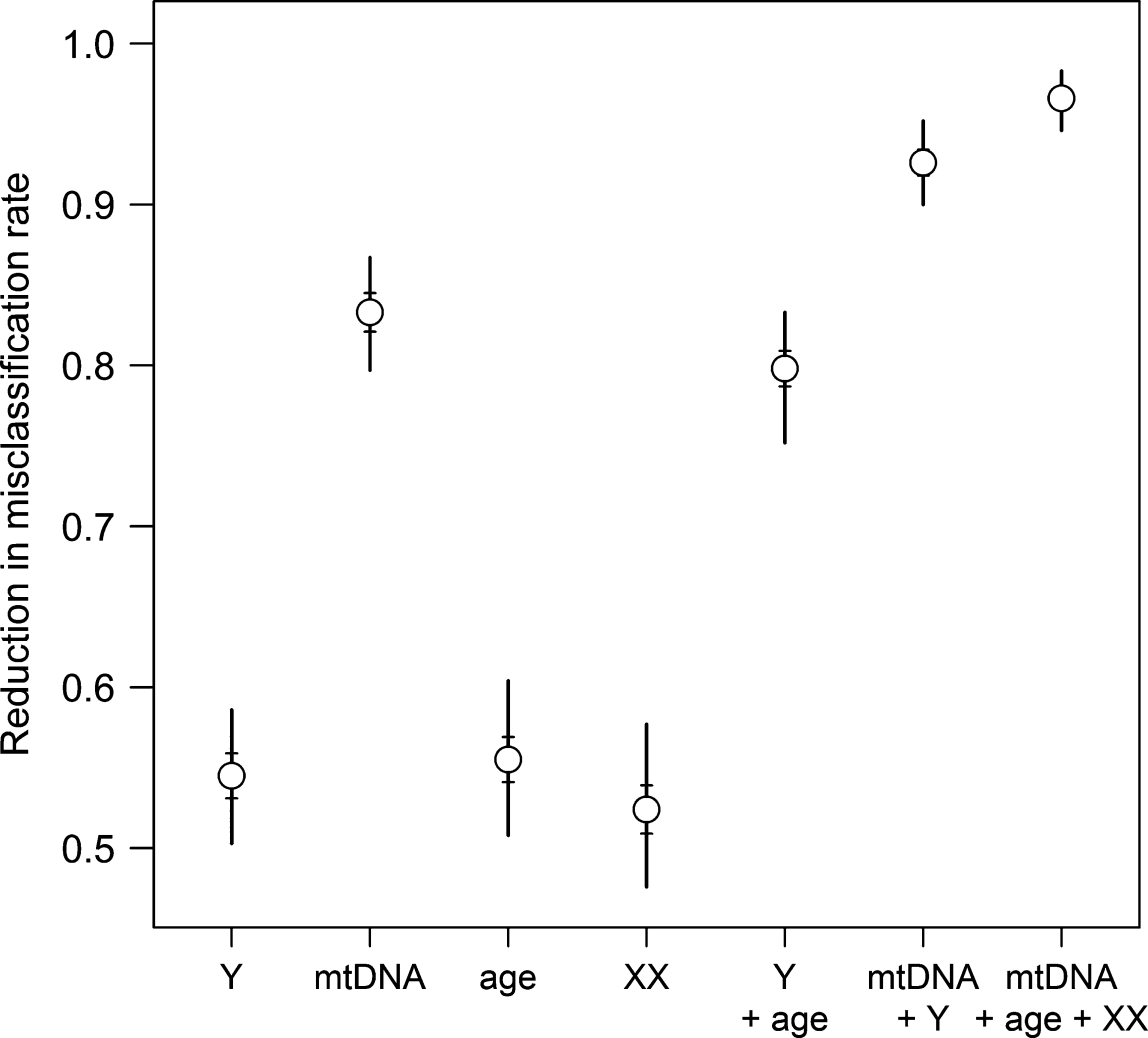
Misclassification rates are reduced when autosomal data are supplemented by other information. Reduction in misclassification rates for 1000 randomized sets of 1000 dyads using the mtDNA and Y‐haplotype frequencies and X‐linked microsatellite allele frequencies of a social group of hamadryas baboons, *Papio hamadryas* (Band 1, Städele et al. 2015). Vertical lines indicate the range, horizontal lines indicate the standard deviation, and circles show the average. Simulations were performed with one Y‐linked microsatellites locus, 13 mtDNA haplotypes, and four X‐linked microsatellites loci (XX). For age, it was assumed for simplicity that two‐thirds of the individuals belonged to one generation and one‐third to another generation and that two individuals of the same generation could not have the supposed relationship.

Fathers and sons have to share Y‐haplotypes, and mothers and offspring have to share mtDNA haplotypes. Fathers and daughters as well as mothers and offspring have to share at least one allele at every locus of the X‐chromosome. Thus, simple comparisons can reveal misclassifications, and the more diverse the marker, the more likely it is that misclassifications are identified because dyads are less likely to share alleles/haplotypes by chance. In a study of hamadryas baboons, we found that misclassification rates can be greatly reduced even using just one Y‐linked microsatellite locus or four X‐linked microsatellite loci with low levels of polymorphism (Fig. 4; Band 1, Städele et al. 2015). In addition to identifying misclassifications, the inclusion of nonautosomal marker data can further improve the resolution of kinship analyses. For example, likelihood ratio values can be calculated for X‐chromosomal genotypes, thus making it for example possible to define cutoffs distinguishing between maternal and paternal siblings if a sufficient number of loci are available (Langergraber et al. 2007). As a further incentive to overcome the challenges of characterization in novel species, it is worth noting that uniparentally inherited markers may also aid in analyses of population structure due to their smaller effective population sizes and help reveal sex‐specific population histories. While mtDNA is maternally inherited for most animals, paternal inheritance of the Y‐chromosome is the norm only in mammals (Sato and Sato 2013). Yet, genetic sex determination via an XY system is found in many fish (Devlin and Nagahama 2002), insects (Sanchez 2008), and reptiles (Modi and Crews 2005). In species with a ZW sex determination system, for which females are the heterogametic sex, shared Z‐chromosomes can facilitate the identification of maternally related male dyads, while shared W‐chromosomes can facilitate the identification of maternally related females. However, species with environmental sex determination and species with a ZW sex determination system lack exclusively paternally inherited markers for the identification of paternal relatives.

In addition to genetic markers, information about age, social status, or group membership may be helpful for identifying false‐positive kinship assignments, for example, by identifying dyads which cannot have the purported relationship due to their relative ages (Kopps et al. 2015; Weinman et al. 2015; Fig. 4). Depending on the life history of the species, age may also be useful to identify the type of a degree of kinship, for example, whether a dyad of second‐degree relatives are grandparent–grandoffspring, half siblings, full avuncular relatives, or double first cousins, which cannot be identified from a small set of autosomal markers. For example, half siblings may be discriminated from grandparent–grandoffspring by the age difference between the individuals in species in which the reproductive life span is shorter than roughly twice the age at first reproduction as, for example, in Hector's dolphins or rock hyraxes (Pacifici et al. 2013). Programs, such as FRANz, allow the use of information about known parentage, sex, birth/beginning of group membership, death/end of group membership, and age at first birth into parentage analyses (Riester et al. 2009).

## Alternatives to Determining Pedigree Kinship

Although it is possible to reliably determine the dyadic kin relationship for some proportions of dyads in a population using a range of different markers, judiciously estimating misclassification rates, and employing demographic data when available, determining dyadic kinship for members of wild populations is challenging. For the investigation of hypotheses which do not require explicit knowledge of kinship, we therefore advocate the use of methods which provide more general and indirect inferences about kinship in a group or population without the necessity of determining the kin relationships of single dyads.

### Who is definitely NOT closely related?

In many studies, researchers seek to determine kinship among individuals to control for possible kin biases while studying other factors that potentially influence social relationships or grouping patterns. In these cases, it might often be sufficient to identify dyads that cannot possibly be close kin and then limit analyses to these dyads. The same is true for breeding programs that aim to identify unrelated dyads among potential wild‐caught founders. Individuals not sharing an mtDNA haplotype cannot be close maternal relatives, individuals not sharing at least one allele at every X‐chromosomal locus cannot be paternal sisters or father–daughter, and males not sharing a Y‐chromosomal haplotype cannot be close paternal relatives. A considerable advantage of this exclusion approach is that one polymorphic marker of each category is sufficient to exclude these categories of kinship, although a larger number and greater variability will lead to the exclusion of more dyads.

### Correlating dyadic relatedness coefficients and other variables

Average dyadic relatedness coefficients can be compared among groups to, for example, make inferences about philopatry and dispersal by comparing average relatedness within groups to average relatedness among groups or average within‐group relatedness values for male and female dyads (Janečka et al. 2007; Li and Merilä 2010; Städele et al. 2015). However, it is often interesting to know whether a certain variable is dependent on relatedness, such as whether more closely related individuals are in closer spatial proximity, have more similar phenotypes, or more often show certain dyadic behaviors. In studies of wild populations for which experimental hypothesis testing is usually not possible, correlational hypothesis testing is used to infer causal relationships.

The Mantel test, which assesses the correlation between two distance matrices, has been used to test for nonrandom correlations between the dyadic relatedness coefficient and other variables, such as spatial distance, acoustic similarity (Lemasson et al. 2011), similarity of odor profiles (Boulet et al. 2009), or association strength and mtDNA sharing (Wiszniewski et al. 2010). Extensions of the simple Mantel test (Mantel 1967), the partial Mantel test (Manly 1986), or multiple regressions on distance matrices (Smouse et al. 1986) allow for testing the correlation between two variables while controlling for one or more other variables. Although Mantel tests have been widely used in ecological studies to assess the relationship between geographic and genetic distance, their statistical appropriateness has been critically discussed, and particularly, the extensions of the simple Mantel test have been criticized in terms of low power and inflated type I error rates (Guillot and Rousset 2013; Legendre et al. 2015). Generally, it seems that simple Mantel tests reveal unbiased results if the assumptions of the test are met, including linearity, homoscedasticity, and a lack of autocorrelation of the permuted variable (Guillot and Rousset 2013). However, the limitations of the Mantel test make it useful only for the investigation of simple hypotheses.

Generalized linear models allow the assessment of more complex hypotheses by testing the influence of several continuous or categorical variables on a response variable including interactions or non‐normal error structures, for example, whether there is an influence of genetic relatedness on spatial proximity and whether this is different for male and female dyads. Data collected from wild populations will rarely correspond to the standard experimental designs in which each individual is part of only one dyad, but instead some or all individuals are part of many dyads. To control for the nonindependence of the data introduced by these multiple “observations”, random effects can be included in (generalized) linear‐mixed models. For dyadic data, this is not always trivial because the individuals within a dyad are often indistinguishable in that they cannot be placed in a meaningful order, such as male and female or aggressor and aggressed (Kenny et al. 2006), and so the assignment of each individual to one of the two random effects for a dyad is arbitrary. A possible solution to this is to repeatedly randomize this assignment and report model results averaged over the randomizations (e.g., Van Leeuwen et al. 2012).

It is important to note that the sample size and the variance in the relatedness composition of the population determine the power of any test correlating dyadic relatedness coefficients with other variables. In particular, wild populations with complex kinship structures may contain a very low proportion of highly related dyads (Csilléry et al. 2006; Van Horn et al. 2008). Furthermore, kin‐biased behaviors might only be expressed toward one type of kin but not another, even though the two kin categories have the same mean genetic relatedness (e.g., parent–offspring and full siblings). Researchers should be aware of this reduced power when interpreting nonsignificant results.

## Future Directions and Challenges

Work in humans aimed at identifying relationships in human pedigree data illustrates the potential of large‐scale data from SNP arrays or whole‐genome sequencing. For example, relatives up to the third degree can be identified with extremely low rates of misclassifications using 500k SNPs (Manichaikul et al. 2010). Relationships can be detected up to the fifth degree with high accuracy using thousands of unlinked SNPs (Kling et al. 2012), and incorporation of linkage information among SNPs may distinguish between different relationships possessing the same degree of relatedness (Kyriazopoulou‐Panagiotopoulou et al. 2011). Stretches of sequence identical by descent inferred from whole‐genome sequence data may resolve relationships up to the fifth degree (Huff et al. 2011; Li et al. 2014). Thus, even distantly related dyads can be accurately classified if a large number of markers, linkage information, or whole‐genome sequence data can be attained.

Programs for pedigree reconstruction should eliminate the need for dyadic relationship classification while at the same time clearly defining the type and not just the degree of the relationship. However, these programs currently do not accommodate complex social systems with nonmonogamy, ungenotyped individuals central to the pedigree, assume sampled individuals are in the same generation, or require large‐scale SNP or whole‐genome sequencing data (Riester et al. 2009; Kirkpatrick et al. 2011; Cussens et al. 2013; He and Eskin 2014; Staples et al. 2014).

Only in the recent years have researchers employed large‐scale cross‐species SNP‐typing approaches, such as SNP chips, which make it possible to genotype large arrays of SNPs for species other than model organisms and commercial species (Pertoldi et al. 2010; Ogden et al. 2012; Hoffman et al. 2013). However, recovery rates for polymorphic SNPs may be low even in closely related species, as in a study in which cross‐amplification of bighorn sheep (*Ovis canadensis*) DNA on a 49,035 loci domestic sheep SNP array (*Ovis aries*), species which diverged ~2.6 Mya, yielded only 561 polymorphic SNPs (1.1%) (Miller et al. 2011). Few studies have used large SNP arrays for low‐quality DNA (Decker et al. 2009). However, efficient semi‐automated smaller‐scale approaches may yield genotypes of ~100 SNPs from low‐quality fecal or hair DNA (Kraus et al. 2015; Norman and Spong 2015).

The potential of next‐generation high‐throughput sequencing to generate large amounts of sequence data from even low‐quality samples, such as ancient DNA, would suggest that large numbers of noninvasive samples could soon be efficiently genotyped or sequenced on a genomic scale. However, demonstrations of efficient low‐cost genome‐level sequencing from noninvasively obtained low‐quality samples are thus far limited in scale (Perry et al. 2010; Chiou and Bergey 2015; Snyder‐Mackler et al. 2016). One recent study used DNA from 62 baboon fecal samples to produce low‐coverage genomes (0.49×) and infer paternity for 27 offspring (Snyder‐Mackler et al. 2016). They estimated a cost of 200 USD/individual for coverage of 1x, approximately twice the amount needed to generate comparably effective microsatellite genotypes for paternity inference. Higher‐coverage genomes may be obtained through the improvements in enrichment of host DNA from fecal samples, although prescreening of DNA samples for the minority containing relatively higher proportions of host DNA remains important (Chiou and Bergey 2015).

Methodologies for efficient and cost‐effective genotyping from low‐quality DNA are only starting to be developed for larger panels of SNPs and whole‐genome sequencing, and particularly for the latter, an enormous amount of know‐how and postprocessing is necessary. Therefore, microsatellites will, at least in the near future, stay the marker of choice for most studies of wild populations for which only low‐quality DNA can be obtained, and determining kinship in these populations will remain challenging.

## Conflict of Interest

None declared.

## Supporting information


**Table S1.** Numbers of microsatellites (STRs) and single‐nucleotide polymorphisms (SNPs) with equal power for kinship analyses.

## References

[ece32346-bib-0001] Almudevar, A. , and E. C. Anderson . 2012 A new version of PRT software for sibling groups reconstruction with comments regarding several issues in the sibling reconstruction problem. Mol. Ecol. Resour. 12:164–178. 21883980 10.1111/j.1755-0998.2011.03061.x

[ece32346-bib-0002] Barelli, C. , K. Matsudaira , T. Wolf , C. Roos , M. Heistermann , K. Hodges , et al. 2013 Extra‐pair paternity confirmed in wild white‐handed gibbons. Am. J. Primatol. 75:1185–1195. 23877831 10.1002/ajp.22180

[ece32346-bib-0003] Barth, M. B. , R. F. A. Moritz , and F. B. Kraus . 2014 The evolution of extreme polyandry in social insects: insights from army ants. PLoS One 9:e105621. 25144731 10.1371/journal.pone.0105621 PMC4140799

[ece32346-bib-0004] Benjamini, Y. , and Y. Hochberg . 1995 Controlling the false discovery rate: a practical and powerful approach to multiple testing. J. R. Stat. Soc. Series B 57:289–300.

[ece32346-bib-0005] Bercovitch, F. B. , and P. S. M. Berry . 2013 Herd composition, kinship and fission‐fusion social dynamics among wild giraffe. Afr. J. Ecol. 51:206–216.

[ece32346-bib-0006] Bérénos, C. , P. A. Ellis , J. G. Pilkington , and J. M. Pemberton . 2014 Estimating quantitative genetic parameters in wild populations: a comparison of pedigree and genomic approaches. Mol. Ecol. 23:3434–3451. 24917482 10.1111/mec.12827 PMC4149785

[ece32346-bib-0007] Berger‐Wolf, T. Y. , S. I. Sheikh , B. DasGupta , M. V. Ashley , I. C. Caballero , W. Chaovalitwongse , et al. 2007 Reconstructing sibling relationships in wild populations. Bioinformatics 23:49–56. 10.1093/bioinformatics/btm219 17646334

[ece32346-bib-0008] Blouin, M. S. 2003 DNA‐based methods for pedigree reconstruction and kinship analysis in natural populations. Trends Ecol. Evol. 18:503–511.

[ece32346-bib-0009] Blouin, M. , M. Parsons , V. Lacaille , and S. Lotz . 1996 Use of microsatellite loci to classify individuals by relatedness. Mol. Ecol. 5:393–401. 8688959 10.1111/j.1365-294x.1996.tb00329.x

[ece32346-bib-0010] Boulet, M. , M. J. E. Charpentier , and C. M. Drea . 2009 Decoding an olfactory mechanism of kin recognition and inbreeding avoidance in a primate. BMC Evol. Biol. 9:281. 19958525 10.1186/1471-2148-9-281 PMC2799416

[ece32346-bib-0011] Bradley, B. , D. Doran‐Sheehy , and D. Lukas . 2004 Dispersed male networks in western gorillas. Curr. Biol. 14:510–513. 15043817 10.1016/j.cub.2004.02.062

[ece32346-bib-0012] Buschiazzo, E. , and N. J. Gemmell . 2010 Conservation of human microsatellites across 450 million years of evolution. Genome Biol. Evol. 2:153–165. 20333231 10.1093/gbe/evq007 PMC2839350

[ece32346-bib-0013] Campbell, N. R. , and S. R. Narum . 2009 Quantitative PCR assessment of microsatellite and SNP genotyping with variable quality DNA extracts. Conserv. Genet. 10:779–784.

[ece32346-bib-0014] Chiou, K. L. , and C. M. Bergey . 2015 FecalSeq: methylation‐based enrichment for noninvasive population genomics from feces. bioRxiv 1–8. doi: 10.1101/032870. 10.1038/s41598-018-20427-9 PMC5792461 29386638

[ece32346-bib-0015] Clutton‐Brock, T. , and B. C. Sheldon . 2010 Individuals and populations: the role of long‐term, individual‐based studies of animals in ecology and evolutionary biology. Trends Ecol. Evol. 25:562–573. 20828863 10.1016/j.tree.2010.08.002

[ece32346-bib-0016] Csilléry, K. , T. Johnson , D. Beraldi , T. Clutton‐Brock , D. Coltman , B. Hansson , et al. 2006 Performance of marker‐based relatedness estimators in natural populations of outbred vertebrates. Genetics 173:2091–2101. 16783017 10.1534/genetics.106.057331 PMC1569738

[ece32346-bib-0017] Cussens, J. , M. Bartlett , E. M. Jones , and N. A. Sheehan . 2013 Maximum likelihood pedigree reconstruction using integer linear programming. Genet. Epidemiol. 37:69–83. 23034892 10.1002/gepi.21686

[ece32346-bib-0018] Decker, J. E. , J. C. Pires , G. C. Conant , S. D. McKay , M. P. Heaton , K. Chen , et al. 2009 Resolving the evolution of extant and extinct ruminants with high‐throughput phylogenomics. Proc. Natl Acad. Sci. USA 106:18644–18649. 19846765 10.1073/pnas.0904691106 PMC2765454

[ece32346-bib-0019] Devlin, R. H. , and Y. Nagahama . 2002 Sex determination and sex differentiation in fish: an overview of genetic, physiological, and environmental influences. Aquaculture 208:191–364.

[ece32346-bib-0020] Dexter, D. , and D. G. Brown . 2013 Fast half‐sibling population reconstruction: theory and algorithms. Algorithms Mol. Biol. 8:20. 23849037 10.1186/1748-7188-8-20 PMC3738158

[ece32346-bib-0021] Dubuc, C. , S. Winters , W. L. Allen , L. J. N. Brent , J. Cascio , D. Maestripieri , et al. 2014 Sexually selected skin colour is heritable and related to fecundity in a non‐human primate. Proc. Biol. Sci. 281:20141602. 25253459 10.1098/rspb.2014.1602 PMC4211451

[ece32346-bib-0022] Ellegren, H. 2003 Levels of polymorphism on the sex‐limited chromosome: a clue to Y from W? BioEssays 25:163–167. 12539242 10.1002/bies.10228

[ece32346-bib-0023] Ellegren, H. 2004 Microsatellites: simple sequences with complex evolution. Nat. Rev. Genet. 5:435–445. 15153996 10.1038/nrg1348

[ece32346-bib-0024] Frère, C. H. , E. Krzyszczyk , E. M. Patterson , S. Hunter , A. Ginsburg , and J. Mann . 2010 Thar she blows! A novel method for DNA collection from cetacean blow. PLoS One 5:1–5. 10.1371/journal.pone.0012299 PMC2928266 20811619

[ece32346-bib-0025] Gay, L. , M. Siol , and J. Ronfort . 2013 Pedigree‐free estimates of heritability in the wild: promising prospects for selfing populations. PLoS One 8:e66983. 23825602 10.1371/journal.pone.0066983 PMC3692515

[ece32346-bib-0026] Glaubitz, J. C. , O. E. Rhodes , and J. A. Dewoody . 2003 Prospects for inferring pairwise relationships with single nucleotide polymorphisms. Mol. Ecol. 12:1039–1047. 12753222 10.1046/j.1365-294x.2003.01790.x

[ece32346-bib-0027] Greminger, M. P. , M. Krützen , C. Schelling , A. Pienkowska‐Schelling , and P. Wandeler . 2010 The quest for Y‐chromosomal markers – methodological strategies for mammalian non‐model organisms. Mol. Ecol. Resour. 10:409–420. 21565040 10.1111/j.1755-0998.2009.02798.x

[ece32346-bib-0028] Guichoux, E. , L. Lagache , S. Wagner , P. Chaumeil , P. Léger , O. Lepais , et al. 2011 Current trends in microsatellite genotyping. Mol. Ecol. Resour. 11:591–611. 21565126 10.1111/j.1755-0998.2011.03014.x

[ece32346-bib-0029] Guillot, G. , and F. Rousset . 2013 Dismantling the Mantel tests. Methods Ecol. Evol. 4:336–344.

[ece32346-bib-0030] Hamilton, W. D. 1964 The genetical evolution of social behaviour I. J. Theor. Biol. 7:1–16. 5875341 10.1016/0022-5193(64)90038-4

[ece32346-bib-0031] Harrison, H. B. , P. Saenz‐Agudelo , S. Planes , G. P. Jones , and M. L. Berumen . 2013 Relative accuracy of three common methods of parentage analysis in natural populations. Mol. Ecol. 22:1158–1170. 23278953 10.1111/mec.12138

[ece32346-bib-0032] Hashimoto, C. , O. Takenaka , and T. Furuichi . 1996 Matrilineal kin relationship and social behavior of wild bonobos (*Pan paniscus*): sequencing the D‐loop region of mitochondrial DNA. Primates 37:305–318.

[ece32346-bib-0033] Hayakawa, S. , and O. Takenaka . 1999 Urine as another potential source for template DNA in polymerase chain reaction (PCR). Am. J. Primatol. 48:299–304. 10402038 10.1002/(SICI)1098-2345(1999)48:4<299::AID-AJP5>3.0.CO;2-G

[ece32346-bib-0034] He, D. , and E. Eskin . 2014 IPED2X: a robust pedigree reconstruction algorithm for complicated pedigrees. J. Bioinform. Comput. Biol. 12:1442007. 25553812 10.1142/S0219720014420074

[ece32346-bib-0035] Hill, W. G. , and B. S. Weir . 2011 Variation in actual relationship as a consequence of Mendelian sampling and linkage. Genet. Res. 93:47–64. 10.1017/S0016672310000480 PMC3070763 21226974

[ece32346-bib-0036] Hoffman, J. I. , M. A. S. Thorne , R. McEwing , J. Forcada , and R. Ogden . 2013 Cross‐amplification and validation of SNPs conserved over 44 million years between seals and dogs. PLoS One 8:1–10. 10.1371/journal.pone.0068365 PMC3712990 23874599

[ece32346-bib-0037] Höss, M. , M. Kohn , S. Pääbo , F. Knauer , and W. Schröder . 1992 Excrement analysis by PCR. Nature 359:199. 1528260 10.1038/359199a0

[ece32346-bib-0038] Huck, M. , E. Fernandez‐Duque , P. Babb , and T. Schurr . 2014 Correlates of genetic monogamy in socially monogamous mammals: insights from Azara's owl monkeys. Proc. Biol. Sci. 281:20140195. 24648230 10.1098/rspb.2014.0195 PMC3973279

[ece32346-bib-0039] Huff, C. D. , D. J. Witherspoon , T. S. Simonson , J. Xing , W. S. Watkins , Y. Zhang , et al. 2011 Maximum‐likelihood estimation of recent shared ancestry (ERSA). Genome Res. 21:768–774. 21324875 10.1101/gr.115972.110 PMC3083094

[ece32346-bib-0040] Janečka, J. E. , T. L. Blankenship , D. H. Hirth , C. William Kilpatrick , M. E. Tewes , and L. I. Grassman . 2007 Evidence for male‐biased dispersal in bobcats *Lynx rufus* using relatedness analysis. Wildl. Biol. 13:38–47.

[ece32346-bib-0041] Jones, A. G. , C. M. Small , K. A. Paczolt , and N. L. Ratterman . 2010 A practical guide to methods of parentage analysis. Mol. Ecol. Resour. 10:6–30. 21564987 10.1111/j.1755-0998.2009.02778.x

[ece32346-bib-0042] Kardos, M. , G. Luikart , and F. W. Allendorf . 2015 Measuring individual inbreeding in the age of genomics: marker‐based measures are better than pedigrees. Heredity 115:63–72. 26059970 10.1038/hdy.2015.17 PMC4815495

[ece32346-bib-0043] Kenny, D. A. , D. A. Kashy , and W. L. Cook . 2006 Dyadic data analysis. Guilford Press, New York, NY.

[ece32346-bib-0044] Kirkpatrick, B. , S. C. Li , R. M. Karp , and E. Halperin . 2011 Pedigree reconstruction using identity by descent. J. Comput. Biol. 18:1481–1493. 22035331 10.1089/cmb.2011.0156

[ece32346-bib-0045] Kling, D. , J. Welander , A. Tillmar , Ø. Skare , T. Egeland , and G. Holmlund . 2012 DNA microarray as a tool in establishing genetic relatedness – current status and future prospects. Forensic Sci. Int. Genet. 6:322–329. 21813350 10.1016/j.fsigen.2011.07.007

[ece32346-bib-0046] König, B. . 1994 Components of lifetime reproductive success in communally and solitary nursing house mice – a laboratory study. Behav. Ecol. Sociobiol. 34:275–283.

[ece32346-bib-0047] Konovalov, D. A. , and D. Heg . 2008 A maximum‐likelihood relatedness estimator allowing for negative relatedness values. Mol. Ecol. Resour. 8:256–263. 21585767 10.1111/j.1471-8286.2007.01940.x

[ece32346-bib-0048] Konovalov, D. A. , C. Manning , and M. T. Henshaw . 2004 Kingroup: a program for pedigree relationship reconstruction and kin group assignments using genetic markers. Mol. Ecol. Notes 4:779–782.

[ece32346-bib-0049] Kopps, A. M. , J. Kang , W. B. Sherwin , and P. J. Palsboll . 2015 How well do molecular and pedigree relatedness correspond, in populations with diverse mating systems, and various types and quantities of molecular and demographic data? G3 5:1815–1826. 26134496 10.1534/g3.115.019323 PMC4555218

[ece32346-bib-0050] Kraus, R. H. S. , B. Cocchiararo , V. Harms , H. Bayerl , R. Kühn , D. W. Förster , et al. 2015 A single‐nucleotide polymorphism‐based approach for rapid and cost‐effective genetic wolf monitoring in Europe based on non‐invasively collected samples. Mol. Ecol. Resour. 15:295–305. 25042673 10.1111/1755-0998.12307

[ece32346-bib-0051] Kyriazopoulou‐Panagiotopoulou, S. , D. K. Haghighi , S. J. Aerni , A. Sundquist , S. Bercovici , and S. Batzoglou . 2011 Reconstruction of genealogical relationships with applications to Phase III of HapMap. Bioinformatics 27:333–341. 10.1093/bioinformatics/btr243 PMC3117348 21685089

[ece32346-bib-0052] Langergraber, K. E. , J. C. Mitani , and L. Vigilant . 2007 The limited impact of kinship on cooperation in wild chimpanzees. Proc. Natl Acad. Sci. USA 104:7786–7790. 17456600 10.1073/pnas.0611449104 PMC1876525

[ece32346-bib-0053] Legendre, P. , M.‐J. Fortin , and D. Borcard . 2015 Should the Mantel test be used in spatial analysis? Methods Ecol. Evol. 6:1239–1247.

[ece32346-bib-0054] Lemasson, A. , K. Ouattara , E. J. Petit , and K. Zuberbühler . 2011 Social learning of vocal structure in a nonhuman primate? BMC Evol. Biol. 11:362. 22177339 10.1186/1471-2148-11-362 PMC3260242

[ece32346-bib-0055] Li, M.‐H. , and J. Merilä . 2010 Genetic evidence for male‐biased dispersal in the Siberian jay (*Perisoreus infaustus*) based on autosomal and Z‐chromosomal markers. Mol. Ecol. 19:5281–5295. 20977509 10.1111/j.1365-294X.2010.04870.x

[ece32346-bib-0056] Li, H. , G. Glusman , C. Huff , J. Caballero , and J. C. Roach . 2014 Accurate and robust prediction of genetic relationship from whole‐genome sequences. PLoS One 9:1–6. 10.1371/journal.pone.0085437 PMC3938395 24586241

[ece32346-bib-0057] Lizé, A. , D. Carval , A. M. Cortesero , S. Fournet , and D. Poinsot . 2006 Kin discrimination and altruism in the larvae of a solitary insect. Proc. Biol. Sci. 273:2381–2386. 16928642 10.1098/rspb.2006.3598 PMC1636088

[ece32346-bib-0058] Lopes, M. S. , F. F. Silva , B. Harlizius , N. Duijvesteijn , P. S. Lopes , S. E. Guimarães , et al. 2013 Improved estimation of inbreeding and kinship in pigs using optimized SNP panels. BMC Genet. 14:92. 24063757 10.1186/1471-2156-14-92 PMC3849284

[ece32346-bib-0060] MacLeod, K. J. , J. F. Nielsen , and T. H. Clutton‐Brock . 2013 Factors predicting the frequency, likelihood and duration of allonursing in the cooperatively breeding meerkat. Anim. Behav. 86:1059–1067.

[ece32346-bib-0061] Manichaikul, A. , J. C. Mychaleckyj , S. S. Rich , K. Daly , M. Sale , and W. M. Chen . 2010 Robust relationship inference in genome‐wide association studies. Bioinformatics 26:2867–2873. 20926424 10.1093/bioinformatics/btq559 PMC3025716

[ece32346-bib-0062] Manly, B. F. J. 1986 Randomization and regression methods for testing for association with geographical, environmental and biological distances between populations. Res. Popul. Ecol. 28:201–218.

[ece32346-bib-0063] Mantel, N. 1967 The detection of disease clustering and a generalized regression approach. Cancer Res. 27:209–220. 6018555

[ece32346-bib-0064] Marshall, T. C. , J. Slate , L. E. Kruuk , and J. M. Pemberton . 1998 Statistical confidence for likelihood‐based paternity inference in natural populations. Mol. Ecol. 7:639–655. 9633105 10.1046/j.1365-294x.1998.00374.x

[ece32346-bib-0065] Mateo, J. M. , and W. G. Holmes . 2004 Cross‐fostering as a means to study kin recognition. Anim. Behav. 68:1451–1459.

[ece32346-bib-0066] Metzger, M. , C. Bernstein , T. S. Hoffmeister , and E. Desouhant . 2010 Does kin recognition and sib‐mating avoidance limit the risk of genetic incompatibility in a parasitic wasp? PLoS One 5:e13505. 20976063 10.1371/journal.pone.0013505 PMC2957437

[ece32346-bib-0067] Miller, J. M. , J. Poissant , J. W. Kijas , and D. W. Coltman . 2011 A genome‐wide set of SNPs detects population substructure and long range linkage disequilibrium in wild sheep. Mol. Ecol. Resour. 11:314–322. 21429138 10.1111/j.1755-0998.2010.02918.x

[ece32346-bib-0068] Milligan, B. G. 2003 Maximum‐likelihood estimation of relatedness. Genetics 163:1153–1167. 12663552 10.1093/genetics/163.3.1153 PMC1462494

[ece32346-bib-0069] Modi, W. S. , and D. Crews . 2005 Sex chromosomes and sex determination in reptiles: Commentary. Curr. Opin. Genet. Dev. 15:660–665. 16214335 10.1016/j.gde.2005.09.009

[ece32346-bib-0070] Morin, P. A. , and D. S. Woodruff . 1992 Paternity exclusion using multiple hypervariable microsatellite loci amplified from nuclear DNA of hair cells Pp. 63–81 *in* Martin R. D., Dixson A. F. and Wickings E. J., eds. Paternity in primates: genetic tests and theories. Basel, S. Karger AG.

[ece32346-bib-0071] Morin, P. A. , G. Luikart , and R. K. Wayne . 2004 SNPs in ecology, evolution and conservation. Trends Ecol. Evol. 19:208–216.

[ece32346-bib-0072] Muralidhar, P. , F. P. De Sá , C. F. B. Haddad , and K. R. Zamudio . 2014 Kin‐bias, breeding site selection and female fitness in a cannibalistic neotropical frog. Mol. Ecol. 23:453–463. 24237705 10.1111/mec.12592

[ece32346-bib-0073] Norman, A. J. , and G. Spong . 2015 Single nucleotide polymorphism‐based dispersal estimates using noninvasive sampling. Ecol. Evol. 5:3056–3065. 26357536 10.1002/ece3.1588 PMC4559049

[ece32346-bib-0074] Ogden, R. , J. Baird , H. Senn , and R. McEwing . 2012 The use of cross‐species genome‐wide arrays to discover SNP markers for conservation genetics: a case study from Arabian and scimitar‐horned oryx. Conserv. Genet. Resour. 4:471–473.

[ece32346-bib-0075] Olsen, J. B. , C. Busack , J. Britt , and P. Bentzen . 2001 The aunt and uncle effect: an empirical evaluation of the confounding influence of full sibs of parents on pedigree reconstruction. J. Hered. 92:243–247. 11447239 10.1093/jhered/92.3.243

[ece32346-bib-0076] O'Reilly, P. T. , and C. C. Kozfkay . 2014 Use of microsatellite data and pedigree information in the genetic management of two long‐term salmon conservation programs. Rev. Fish Biol. Fisheries 24:819–848.

[ece32346-bib-0077] Pacifici, M. , L. Santini , M. Di Marco , D. Baisero , L. Francucci , G. Grottolo Marasini , et al. 2013 Generation length for mammals. Nat. Conserv. 5:89–94.

[ece32346-bib-0078] Pearce, J. M. , R. L. Fields , and K. T. Scribner . 1997 Nest materials as a source of genetic data for avian ecological studies. J. Field Ornithol. 68:471–481.

[ece32346-bib-0079] Perry, G. H. , J. C. Marioni , P. Melsted , and Y. Gilad . 2010 Genomic‐scale capture and sequencing of endogenous DNA from feces. Mol. Ecol. 19:5332–5344. 21054605 10.1111/j.1365-294X.2010.04888.x PMC2998560

[ece32346-bib-0080] Pertoldi, C. , J. M. Wójcik , M. Tokarska , A. Kawałko , T. N. Kristensen , V. Loeschcke , et al. 2010 Genome variability in European and American bison detected using the BovineSNP50 BeadChip. Conserv. Genet. 11:627–634.

[ece32346-bib-0081] Rajakaruna, R. S. , J. A. Brown , K. H. Kaukinen , and K. M. Miller . 2006 Major histocompatibility complex and kin discrimination in Atlantic salmon and brook trout. Mol. Ecol. 15:4569–4575. 17107483 10.1111/j.1365-294X.2006.03113.x

[ece32346-bib-0082] Rasmuson, M. 1993 Variation in genetic identity within kinships. Heredity 70(Pt 3):266–268. 8458731 10.1038/hdy.1993.38

[ece32346-bib-0083] Riester, M. , P. F. Stadler , and K. Klemm . 2009 FRANz: reconstruction of wild multi‐generation pedigrees. Bioinformatics 25:2134–2139. 19202194 10.1093/bioinformatics/btp064 PMC2722992

[ece32346-bib-0120] Sanchez, L. 2008 Sex‐determining mechanisms in insects. Int. J. Dev. Biol. 52:837–856. 18956315 10.1387/ijdb.072396ls

[ece32346-bib-0084] Sanderson, J. L. , J. Wang , E. I. K. Vitikainen , M. A. Cant , and H. J. Nichols . 2015 Banded mongooses avoid inbreeding when mating with members of the same natal group. Mol. Ecol. 24:3738–3751. 26095171 10.1111/mec.13253 PMC5008155

[ece32346-bib-0085] Santure, A. W. , J. Stapley , A. D. Ball , T. R. Birkhead , T. Burke , and J. Slate . 2010 On the use of large marker panels to estimate inbreeding and relatedness: empirical and simulation studies of a pedigreed zebra finch population typed at 771 SNPs. Mol. Ecol. 19:1439–1451. 20149098 10.1111/j.1365-294X.2010.04554.x

[ece32346-bib-0086] Sato, M. , and K. Sato . 2013 Maternal inheritance of mitochondrial DNA by diverse mechanisms to eliminate paternal mitochondrial DNA. Biochim. Biophys. Acta 1833:1979–1984. 23524114 10.1016/j.bbamcr.2013.03.010

[ece32346-bib-0087] Sheikh, S. I. , T. Y. Berger‐Wolf , M. V. Ashley , I. C. Caballero , W. Chaovalitwongse , and B. DasGupta . 2008 Error tolerant sibship reconstruction in wild populations. Comput. Syst. Bioinformatics Conf. 7:273–284. 19642287

[ece32346-bib-0088] Silk, J. B. , J. Altmann , and S. C. Alberts . 2006 Social relationships among adult female baboons (*Papio cynocephalus*) I. Variation in the strength of social bonds. Behav. Ecol. Sociobiol. 61:183–195.

[ece32346-bib-0089] Skaug, H. J. , M. Bérubé , and P. J. Palsbøll . 2010 Detecting dyads of related individuals in large collections of DNA‐profiles by controlling the false discovery rate. Mol. Ecol. Resour. 10:693–700. 21565074 10.1111/j.1755-0998.2010.02833.x

[ece32346-bib-0090] Smith, K. , S. C. Alberts , and J. Altmann . 2003 Wild female baboons bias their social behaviour towards paternal half‐sisters. Proc. Biol. Sci. 270:503–510. 12641905 10.1098/rspb.2002.2277 PMC1691261

[ece32346-bib-0091] Smouse, P. E. , J. C. Long , and R. R. Sokal . 1986 Multiple regression and correlation extensions of the mantel test of matrix correspondence. Syst. Zool. 35:627.

[ece32346-bib-0092] Snyder‐Mackler, N. , W. H. Majoros , M. L. Yuan , A. O. Shaver , J. B. Gordon , G. H. Kopp , et al. 2016 Efficient genome‐wide sequencing and low coverage pedigree analysis from non‐invasively collected samples. Genetics 203:699–714. 27098910 10.1534/genetics.116.187492 PMC4896188

[ece32346-bib-0093] Speed, D. , and D. J. Balding . 2014 Relatedness in the post‐genomic era: is it still useful? Nat. Rev. Genet. 16:33–44. 25404112 10.1038/nrg3821

[ece32346-bib-0094] Städele, V. , V. Van Doren , M. Pines , L. Swedell , and L. Vigilant . 2015 Fine‐scale genetic assessment of sex‐specific dispersal patterns in a multilevel primate society. J. Hum. Evol. 78:103–113. 25466516 10.1016/j.jhevol.2014.10.019

[ece32346-bib-0095] Städele, V. , M. Pines , L. Swedell , and L. Vigilant . 2016 The ties that bind: maternal kin bias in a multilevel primate society despite natal dispersal by both sexes. Am. J. Primatol. 78:731–744. 26890431 10.1002/ajp.22537

[ece32346-bib-0096] Staples, J. , D. Qiao , M. H. Cho , E. K. Silverman , D. A. Nickerson , and J. E. Below . 2014 PRIMUS: rapid reconstruction of pedigrees from genome‐wide estimates of identity by descent. Am. J. Hum. Genet. 95:553–564. 25439724 10.1016/j.ajhg.2014.10.005 PMC4225580

[ece32346-bib-0097] Stiver, K. A. , S. H. Wolff , and S. H. Alonzo . 2012 Adoption and cuckoldry lead to alloparental care in the tessellated darter (*Etheostoma olmstedi*), a non‐group‐living species with no evidence of nest site limitation. Behav. Ecol. Sociobiol. 66:855–864.

[ece32346-bib-0098] Taberlet, P. , and G. Luikart . 1999 Non‐invasive genetic sampling and individual identification. Biol. J. Linn. Soc. 68:41–55.

[ece32346-bib-0099] Thomas, S. C. , and W. G. Hill . 2002 Sibship reconstruction in hierarchical population structures using Markov chain Monte Carlo techniques. Genet. Res. 79:227–234. 12220130 10.1017/s0016672302005669

[ece32346-bib-0100] Thompson, E. A. , and T. R. Meagher . 1987 Parental and sib likelihoods in genealogy reconstruction. Biometrics 43:585–600. 3663818

[ece32346-bib-0101] Van Horn, R. C. , J. Altmann , and S. C. Alberts . 2008 Can't get there from here: inferring kinship from pairwise genetic relatedness. Anim. Behav. 75:1173–1180.

[ece32346-bib-0102] Van Leeuwen, E. J. C. , K. A. Cronin , D. B. M. Haun , R. Mundry , and M. D. Bodamer . 2012 Neighbouring chimpanzee communities show different preferences in social grooming behaviour. Proc. Biol. Sci. 279:4362–4367. 22933372 10.1098/rspb.2012.1543 PMC3479803

[ece32346-bib-0103] Van Noordwijk, M. A. , N. Arora , E. P. Willems , L. P. Dunkel , R. N. Amda , N. Mardianah , et al. 2012 Female philopatry and its social benefits among *Bornean orangutans* . Behav. Ecol. Sociobiol. 66:823–834.

[ece32346-bib-0104] Viblanc, V. A. , C. M. Arnaud , F. S. Dobson , and J. O. Murie . 2010 Kin selection in Columbian ground squirrels (*Urocitellus columbianus*): littermate kin provide individual fitness benefits. Proc. Biol. Sci. 277:989–994. 19939839 10.1098/rspb.2009.1960 PMC2842772

[ece32346-bib-0105] Vigilant, L. , J. Roy , B. J. Bradley , C. J. Stoneking , M. M. Robbins , and T. S. Stoinski . 2015 Reproductive competition and inbreeding avoidance in a primate species with habitual female dispersal. Behav. Ecol. Sociobiol. 69:1163–1172.

[ece32346-bib-0106] Villarreal, X. , J. Bricker , H. Reinert , L. Gelbert , and L. Bushar . 1996 Isolation and characterization of microsatellite loci for use in population genetic analysis in the timber rattlesnake, *Crotalus horridus* . J. Hered. 87:152–155. 8830093 10.1093/oxfordjournals.jhered.a022973

[ece32346-bib-0107] Visscher, P. M. , S. E. Medland , M. A. R. Ferreira , K. I. Morley , G. Zhu , B. K. Cornes , et al. 2006 Assumption‐free estimation of heritability from genome‐wide identity‐by‐descent sharing between full siblings. PLoS Genet. 2:e41. 16565746 10.1371/journal.pgen.0020041 PMC1413498

[ece32346-bib-0108] Visscher, P. M. , G. Hemani , A. E. Vinkhuyzen , G.‐B. Chen , S. H. Lee , N. R. Wray , et al. 2014 Statistical power to detect genetic (co) variance of complex traits using SNP data in unrelated samples. PLoS Genet. 10:e1004269. 24721987 10.1371/journal.pgen.1004269 PMC3983037

[ece32346-bib-0109] Wang, J. 2004 Sibship reconstruction from genetic data with typing errors. Genetics 166:1963–1979. 15126412 10.1534/genetics.166.4.1963 PMC1470831

[ece32346-bib-0110] Wang, J. 2012 Computationally efficient sibship and parentage assignment from multilocus marker data. Genetics 191:183–194. 22367033 10.1534/genetics.111.138149 PMC3338259

[ece32346-bib-0111] Wang, J. 2015 Pedigrees or markers: which are better in estimating relatedness and inbreeding coefficient? Theor. Popul. Biol. 107:4–13. 26344786 10.1016/j.tpb.2015.08.006

[ece32346-bib-0112] Wang, J. , and A. W. Santure . 2009 Parentage and sibship inference from multilocus genotype data under polygamy. Genetics 181:1579–1594. 19221199 10.1534/genetics.108.100214 PMC2666522

[ece32346-bib-0113] Weinman, L. R. , J. W. Solomon , and D. R. Rubenstein . 2015 A comparison of single nucleotide polymorphism and microsatellite markers for analysis of parentage and kinship in a cooperatively breeding bird. Mol. Ecol. Resour. 15:502–511. 25224810 10.1111/1755-0998.12330

[ece32346-bib-0114] Widdig, A. 2013 The impact of male reproductive skew on kin structure and sociality in multi‐male groups. Evol. Anthropol. 22:239–250. 24166924 10.1002/evan.21366

[ece32346-bib-0115] Widdig, A. , D. Langos , and L. Kulik . 2016 Sex differences in kin bias at maturation: male rhesus macaques prefer paternal kin prior to natal dispersal. Am. J. Primatol. 78:78–91. 25810077 10.1002/ajp.22401

[ece32346-bib-0116] Wikberg, E. C. , K. M. Jack , F. A. Campos , L. M. Fedigan , A. Sato , M. L. Bergstrom , et al. 2014 The effect of male parallel dispersal on the kin composition of groups in white‐faced capuchins. Anim. Behav. 96:9–17.

[ece32346-bib-0117] Wiszniewski, J. , D. Lusseau , and L. M. Möller . 2010 Female bisexual kinship ties maintain social cohesion in a dolphin network. Anim. Behav. 80:895–904.

