# Supplementary material for: Strategies for determining kinship in wild populations using genetic data
Source: Ecol Evol. 2016 Jul 29;6(17):6107–20. doi: 10.1002/ece3.2346 (PMC5016635; doi:10.1002/ece3.2346)
Supplement: Supplementary file 1 — Table S1. Numbers of microsatellites (STRs) and single‐nucleotide polymorphisms (SNPs) with equal power for kinship analyses. [file ECE3-6-6107-s001.docx]

Supporting Information

**Strategies for determining kinship in wild populations using genetic data**

*Ecology and Evolution*

**Table S1** Numbers of microsatellites (STRs) and single-nucleotide polymorphisms (SNPs)

with equal power for kinship analyses

| Number of STRs | Number of SNPs | Reference |
| --- | --- | --- |
| 1 | 10 | Wang & Santure 2009 |
| 6 | 32 | Glaubitz *et al.* 2003 |
| 10 | 43 | Herráez *et al.* 2005 |
| 10 | 60 | Rohrer *et al.* 2007 |
| 11 | 80 | Hauser *et al.* 2011 |
| 13 | 63 | Sellars *et al.* 2014 |
| 13 | 59 | Sellars *et al.* 2014 |
| 14 | 100 | Morin *et al.* 2004 |
| 15 | 102 | Weinman *et al.* 2015 |
| 16 | 106 | Telfer *et al.* 2015 |
| 17 | 95 | Steele *et al.* 2013 |
| 18 | 100 | Glaubitz *et al.* 2003 |

**References**

Glaubitz, J.C., Rhodes, O.E. & Dewoody, J.A. (2003). Prospects for inferring pairwise relationships with single nucleotide polymorphisms. *Molecular Ecology*, **12**, 1039–1047.

Hauser, L., Baird, M., Hilborn, R., Seeb, L.W. & Seeb, J.E. (2011). An empirical comparison of SNPs and microsatellites for parentage and kinship assignment in a wild sockeye salmon (*Oncorhynchus nerka*) population. *Molecular Ecology Resources*, **11**, 150–61.

Herráez, D.L., Schäfer, H., Mosner, J., Fries, H.R. & Wink, M. (2005). Comparison of microsatellite and single nucleotide polymorphism markers for the genetic analysis of a galloway cattle population. *Zeitschrift fur Naturforschung - Section C*, **60**, 637–643.

Morin, P. a., Luikart, G. & Wayne, R.K. (2004). SNPs in ecology, evolution and conservation. *Trends in Ecology and Evolution*, **19**, 208–216.

Rohrer, G.A., Freking, B.A. & Nonneman, D. (2007). Single nucleotide polymorphisms for pig identification and parentage exclusion. *Animal Genetics*, **38**, 253–258.

Sellars, M.J., Dierens, L., Mcwilliam, S., Little, B., Murphy, B., Coman, G.J., Barendse, W. & Henshall, J. (2014). Comparison of microsatellite and SNP DNA markers for pedigree assignment in Black Tiger shrimp, *Penaeus monodon*. *Aquaculture Research*, **45**, 417–426.

Steele, C., Anderson, E., Ackerman, M., Hess, M., Campbell, N., Narum, S. & Campbell, M. (2013). A validation of parentage-based tagging using hatchery steelhead in the Snake River basin. *Journal of Fisheries*, **1054**, 1046–1054.

Telfer, E.J., Stovold, G.T., Li, Y., Silva-Junior, O.B., Grattapaglia, D.G. & Dungey, H.S. (2015). Parentage Reconstruction in *Eucalyptus nitens* Using SNPs and Microsatellite Markers: A Comparative Analysis of Marker Data Power and Robustness. *Plos One*, **10**, e0130601.

Wang, J. & Santure, A.W. (2009). Parentage and sibship inference from multilocus genotype data under polygamy. *Genetics*, **181**, 1579–1594.

Weinman, L.R., Solomon, J.W. & Rubenstein, D.R. (2015). A comparison of single nucleotide polymorphism and microsatellite markers for analysis of parentage and kinship in a cooperatively breeding bird. *Molecular Ecology Resources*, **15**, 502–511.
